# Different significance of HRCT and FDG-PET/CT to predict lymph node status between patients with clinical stage IA lung adenocarcinoma and squamous cell carcinoma

**DOI:** 10.1186/1749-8090-10-S1-A332

**Published:** 2015-12-16

**Authors:** Yasuhiro Tsutani, Yoshihiro Miyata, Takeshi Mimura, Masaoki Ito, Yuichiro Kai, Atsushi Kagimoto, Haruhiko Nakayama, Sakae Okumura, Masahiro Yoshimura, Morihito Okada

**Affiliations:** 1 Department of Surgical Oncology, Hiroshima University, Hiroshima, Japan; 2 Department of Thoracic Surgery, Kanagawa Cancer Center, Yokohama, Japan; 3 Department of Thoracic Surgery, Cancer Institute Hospital, Tokyo, Japan; 4 Department of Thoracic Surgery, Hyogo Cancer Center, Akashi, Japan

## Background/Introduction

True node-negative small sized non-small cell lung cancers are optimal candidates for sublobar resection without systematic lymph node dissection.

## Aims/Objectives

The purpose of this study is to identify the predictive factors of true node-negative clinical stage IA non-small cell lung cancer.

## Method

A multicenter database of patients with completely resected clinical stage IA lung adenocarcinoma (n = 502) or squamous cell carcinoma (n = 100) was retrospectively analyzed. The relationship between lymph node status and preoperative factors such as tumor size on HRCT and maximum standardized uptake value (SUVmax) on FDG-PET/CT were examined.

## Results

Multivariate analyses revealed that solid tumor size on HRCT (Odds ratio (OR), 1.42; p < 0.001) or SUVmax on FDG-PET/CT (OR, 1.04; p = 0.049) was identified as an independent predictor of lymph node metastasis in patients with lung adenocarcinoma. The predictive criteria of node-negative lung adenocarcinoma were solid tumor size <0.8 cm or SUVmax <1.5. Among patients who met the node-negative criteria, recurrence-free survival at 5 years was not significantly different between those who underwent lobectomy (96.0%) and those who underwent sublobar resection (97.2%). In patients with squamous cell carcinoma of the lung, no independent predictive factors for lymph node metastasis were identified in univariate or multivariate analysis.

## Discussion/Conclusion

Either solid tumor size on HRCT or SUVmax on FDG-PET/CT was a significant independent predictor of nodal status in clinical stage IA lung adenocarcinoma. The node-negative criteria of solid tumor size <0.8 cm or SUVmax <1.5 are helpful for choosing candidates for sublobar resection without systematic lymphadenectomy. In patients with clinical stage IA lung squamous cell carcinoma, systematic lymphadenectomy is advisable.

